# Voting at Home Is Associated with Lower Cortisol than Voting at the Polls

**DOI:** 10.1371/journal.pone.0135289

**Published:** 2015-09-03

**Authors:** Jayme Neiman, Karl Giuseffi, Kevin Smith, Jeffrey French, Israel Waismel-Manor, John Hibbing

**Affiliations:** 1 Department of Political Science, University of Northern Iowa, Cedar Falls, Iowa, United States of America; 2 Department of Political Science, University of Nebraska-Lincoln, Lincoln, Nebraska, United States of America; 3 Department of Psychology, University of Nebraska-Omaha, Omaha, Nebraska, United States of America; 4 School of Political Sciences, University of Haifa, Mount Carmel, Haifa, Israel; Cinvestav-Merida, MEXICO

## Abstract

Previous research finds that voting is a socially stressful activity associated with increases in cortisol levels. Here we extend this research by investigating whether different voting modalities have differential effects on the stress response to voting. Results from a field experiment conducted during the 2012 presidential elections strongly suggest that traditional “at the polls” voting is more stressful, as measured by increases in cortisol levels, than voting at home by mail-in ballot or engaging in comparable non-political social activities. These findings imply that increased low-stress voting options such as mail-in ballots may increase political participation among individuals who are sensitive to social stressors.

## Introduction

Several recent studies suggest a correlation between political participation and the functioning of the neuroendocrine stress system. Specifically, the basal activity and stress-response of the hypothalamic-pituitary-adrenal (HPA) axis have been assessed by taking individual-level cortisol assays and correlating the results with various aspects of voting [1–4]. Cortisol is a glutocorticoid that helps regulate the HPA axis and is well-known to be associated with stress response (cortisol is sometimes referred to as “the stress hormone”). That cortisol levels co-vary with political participation makes a good deal of sense; differences in HPA-axis function are known to co-vary with a wide range of non-political processes that are social, affective and require making choices [5–7]. By definition, the traditional notion of voting—casting a ballot in-person at a designated polling place—is a social act that requires decision-making. It is well known that for most people political engagement is an emotional experience [8].

The hypothesis that cortisol will systematically co-vary with political participation is already backed by considerable empirical evidence. For example, Waismel-Manor, Ifergane and Cohen compared cortisol levels for voters on election versus non-election days and found those levels were up to five times higher on election days [1]. French et al. reported baseline cortisol levels predict voting participation, even after controlling for variables well-known to predict voting and baseline cortisol levels [2]. Stanton et al. found election-day cortisol levels for supporters of a losing candidate increased when the election results were announced [3]. Similarly, Blanton et al. reported that cortisol levels spiked when supporters of a presidential candidate watched news coverage of his electoral loss [9].

Although these studies consistently demonstrate a link between variation in cortisol levels and political engagement, it is unclear if the link between voting and cortisol levels is a product of the act of voting, the modality of voting, or simply the social (as opposed to the purely political) aspect of voting. Is it going to a public polling place that is stressful? Would alternate, at-home voting options reduce such stress? Is there anything about this stress response that is unique to political participation or would any non-political social interaction comparable to voting (i.e. making a choice) trigger a similar response? These are potentially important questions, especially if lowering the social stress of voting implies even a marginal shift in voting probabilities for those more sensitive to such stressors. In this paper we seek to address these questions by experimentally comparing the impact on cortisol levels of voting in a polling place, voting at home with a mail-in ballot, and engaging in a non-political social interaction.

Specifically, we use a randomized field experiment conducted during the 2012 presidential election that randomly assigned voters to vote either at home, at the polls, or to make a minor purchase at a convenience store. This analytical procedure of randomly assigning subjects to different voting modalities in a real-world election, to our knowledge, has never been attempted before (this project was approved by the pertinent human subjects review board; IRB #2012–0912839). We have the opportunity to establish whether the stress of political participation is a purely social stress tied to voting modality and social interaction or a uniquely political stress tied to making a political choice affecting the lives of others.

## Methods and Materials

### Sample

In the summer of 2010, we retained the services of a professional survey organization to draw a random sample (N = 343) of the voting age population residing within easy driving distance of our lab in a mid-sized U.S. city in the Midwest. Using an appropriate mix of landline and cellphone numbers, a group was identified and that group’s characteristics are reasonably representative of the overall population (AAPOR RR1 = 26 percent: 54 percent female, mean age of 45, modal family income in the $40,000–60,000 category, with 55 percent having at least some college education). In exchange for a participation fee of $50, these individuals agreed to travel to the lab to complete a lengthy computer survey of their personality traits, political preferences, and sociodemographic characteristics, and to engage in selected physiological tests. Given the amount of information collected on these individuals, they were intended to serve as a recruitment pool from which smaller samples could be drawn to engage in more concentrated and perhaps demanding investigations such as those involving neuroimaging and endocrinology. This same subject pool was used for a separate project that found a suppressing effect of stress (baseline cortisol levels) on general voting participation [2].

In the fall of 2012 we asked the same survey organization to re-contact a sample of the original 343 in hopes of securing the participation of a smaller group of participants for this study on voting modalities and stress. Our target N was approximately 40 individuals for each experimental condition. A randomly selected portion of this original group was used in an earlier cortisol-based project, and 70 of these individuals were able to participate again. We asked the survey organization to recruit 75–80 more individuals from the original group with the caveats that those who ultimately participated were registered voters who intended to vote in the 2012 presidential election. The current project was designed to avoid manipulating electoral participation, which is why an expressed intention to vote in the 2012 presidential election was a requirement for inclusion in our sample. Commitments were eventually obtained from 137 individuals (in exchange for the promise of $50 upon completion).

Table 1 presents descriptive statistics for the socio-demographics of the sample used in this study along with comparison means from the full recruitment pool they were drawn from. These suggest that our sample is very similar to the recruitment pool on these measures. It is slightly more female, has a mean age difference of roughly two years, and is virtually identical on our income and education measures (income was measured on a 6-point scale where 1 = below $20,000 and 6 = more than $100,000, education was measured on a 9-point scale where 1 = less than high school, 9 = professional degree/Phd). Basic descriptive statistics, in short, suggest the sample for this study looks very much like the randomly selected pool of adults from which it was drawn.

**Table 1:** Characteristics of Study Sample and Recruitment Pool.

Variable	Sample Mean (N = 137)	Sample Std. Dev (N = 137)	Sample Min. (N = 137)	Sample Max. (N = 137)	Recruitment Pool Mean Characteristics (N = 340)
**Gender (Male = 1)**	0.41	0.49	0.00	1.00	.46
**Age**	47.6	12.91	19.00	65.00	45.6
**Income**	3.76	1.65	1.00	6.00	3.6
**Education**	5.81	1.62	1.00	9.00	5.7

It should be noted that there is one potential source of selection bias for our study that we cannot fully account for: motivation. Subjects for this study were not only willing participants who indicated they were committed to voting in the presidential election, they were also willing to be flexible in the modality of voting they used. In short, they are clearly a group willing to participate in more than politics. That trait, however, likely makes for a conservative test of our hypotheses. Simply put, if we find differences in stress responses to voting amongst people who are willing participators, it is reasonable to think that larger difference may be found in a sample that included less-willing participators and non-participators.

### Method

The 137 participants provided an opportunity to conduct a randomized field experiment because all agreed to be flexible in how and when they voted. This made it possible to randomly assign participants into one of three groups. The first group was the public voting group—individuals who agreed to vote at the polls at approximately 7:00 pm on November 6, 2012. The second group was the vote-at-home group—individuals who agreed to vote absentee by marking their ballots while in their own home at approximately 7:00 pm on October 30, 2012, just a few days before the election and in time for the mailed ballot to be received by the deadline so that it could be counted.

The third and final group was our control. The control group was assigned to vote in whatever fashion they wanted (absentee or public) as long as they did it before 3:00 pm on November 6. At approximately 7:00 p.m. on that day they agreed to engage in a social behavior that was selected to be similar in key aspects to going to the polls, but not actually involve voting. Specifically, participants in this group were instructed to leave their home, go to a local convenience store and make a small purchase of a non-food item. The idea here was to assign a behavioral task that was similar to going to the polls in the following ways: it involved leaving the home, driving to a public place in the neighborhood, seeing and interacting with other people, making a decision and perhaps being required to wait in line. While no activity perfectly mirrors going to the polls, we reasoned this constituted a reasonable behavioral proxy, especially as in the state where the experiment was conducted neither convenience stores nor polling places are particularly crowded or chaotic. It is rare to wait longer than a few minutes to cast a ballot or make a convenience store purchase. On these dimensions the convenience store control mirrors polling places reasonably well. For all three groups, cortisol samples were taken immediately prior to, and then again shortly after, the assigned activity.

The central aim of our experimental design is to disentangle the stress-inducing effects, as measured by cortisol changes, of making a decision, making a voting decision, and going somewhere outside of one’s domicile to make a voting decision. The hypotheses are straightforward. If stress is generated by a voting decision regardless of the modality by which that decision is rendered, we should see increases in cortisol levels for the “vote at the polls” and the “vote at home” groups but not the “go to a convenience store” (control) group. If stress is generated by leaving home in order to travel to a nearby public place regardless of the purpose of leaving home, we should see increases in cortisol levels for the “vote at the polls” and the “go to a convenience store” groups but not the “vote at home” group. If stress is generated only by the combination of voting AND leaving home in order to travel to a nearby public place, we should see an increase in cortisol levels for only the “vote at the polls” group.

### Materials

Participants were instructed to provide saliva samples both before and after they engaged in the target act to which they had been randomly assigned. They did so with SciMart Salivettes, which use a procedure that requires the participants to chew on a roll-shaped synthetic saliva collector before placing that collector in a sealed tube. This technique does not require participants to generate large quantities of saliva. Instead, participants simply chew on the saliva collector until it is moist. Participants were asked to provide two samples: one taken at approximately 6:30pm, shortly before the target act (which was to be performed as close to 7:00pm as possible) and the other taken at approximately 7:30 pm, shortly after the target act. Previous research demonstrates that cortisol typically peaks about 20–30 minutes after a stressor [10,11]. Participants were instructed to complete the survey (S1 File: Survey Items) immediately after producing the second saliva sample. They were then required to send in the two samples, along with their completed survey in a provided postage paid envelope. The methodology of asking participants to produce and mail in their saliva samples has been employed successfully in previous studies [12,13].

Once we received these samples, they were assayed for cortisol concentration by enzyme immunoassay (EIA) which we describe in the S2 File: Methods. The difference between cortisol levels in the samples taken before and after the target act is our central indicator of physiological stress levels. The requirement that all target acts occur at approximately 7:00 pm stems primarily from the fact that cortisol levels follow a clear diurnal rhythm. On average, cortisol levels start high, drop fairly sharply during the morning hours, and then drop more slowly during the afternoon and evening. Controlling for sample collection time minimizes these potentially confounding effects. Cortisol levels can also be affected by things like smoking, alcohol, anti-depressant use, pregnancy, birth control medications, and exercise. Participants were asked about these in the survey, and tests revealed no mean differences between the conditions on any of the variables. To minimize further confounds, we also instructed participants to avoid eating and drinking anything but water from 5:00 pm until after the post-target act sample had been placed in the tube and the survey completed (at approximately 8:00 pm).

One hundred and thirty three of the 137 individuals who agreed to complete the study provided survey and usable saliva samples, a falloff rate that was (pleasingly) smaller than we anticipated. These 133 were distributed fairly evenly across groups: 47 in the “vote at the polls” group; 42 in the “vote at home” group; and 44 in the “go to the convenience store” group (total N = 133).

## Results

Cortisol change is our central variable of interest and it was derived by computing the difference between cortisol levels in nanograms per milliliter (ng/ml) taken before the target act and cortisol levels taken after the target act. The pre-target act cortisol level was subtracted from the post-target act cortisol level so higher, positive values indicate an increase in cortisol levels. The base expectation is that if the assigned behavior induces no stress response, cortisol levels should not change or slightly decrease as hormone levels naturally drop as part of cortisol’s diurnal rhythm. Mean cortisol differences were fairly normally distributed, but there were a handful of outliers so we winsorized the data at the 95^th^ and 5^th^ percentiles to minimize their influence on the results.

Before examining whether cortisol changes differed by experimental group, we first checked for a range of potential equivalency confounds to see whether any experimental condition systematically differed in a way that might influence our results. These consisted of a series of ANOVAs to examine mean differences across groups. We found no significant differences between groups in age, income, education, gender, Postitive and Negative Affect Scale (PANAS) scores, and, most importantly, pre-treatment cortisol levels. PANAS is designed to measure self-reported and non-time-sensitive negative and positive affect [14, 15]. We include it here because negative affect, especially, is an individual trait that has been associated with differential cortisol responses to stress stimuli [4]. Accordingly, we use the five negative affect items taken from the 10-item PANAS [14]. These items ask “do you normally feel upset; hostile; ashamed; nervous; afraid,” and the available options are “a great deal” (5); “a lot” (4); “a moderate amount” (3); “a little” (2); or “not at all” (1). These items have reasonable internal consistency (alpha = .68) and factor analysis revealed a single dimension that accounted for 74% of the average variation between the items. We report results using a simple additive scale of PANAS items. Results of our equivalency checks are reported in Table 2. The lack of any significant differences on these variables suggest that random assignment is, as the experimental design intends, controlling for any potential initial equivalence issues associated with our key dependent variable and thus can attribute any changes in cortisol levels to our experimental manipulations.

**Table 2:** Testing for Equivalency Differences Between Experimental Conditions.

Variable	Estimated Means (Control Condition)	Estimated Means (Absentee Condition)	Estimated Means (Poll Condition)	Difference Test
**Age**	47.40	46.80	48.48	F(2,134) = .19, p>.80
**Income**	3.60	3.81	3.85	F(2,134) = .27, p>.70
**Education**	5.88	6.00	5.54	F(2,134) = .99, p>.30
**PANAS**	5.32	5.11	5.25	F(2,134) = .19, p>.80
**Gender (Female)**	58.97	60.98	60.00	df = 2, Chi-square = .0334, p>.50
**Pre Cortisol Levels**	3.21	3.74	3.50	F(2,134) = .68, p>.50

**Fig 1:**
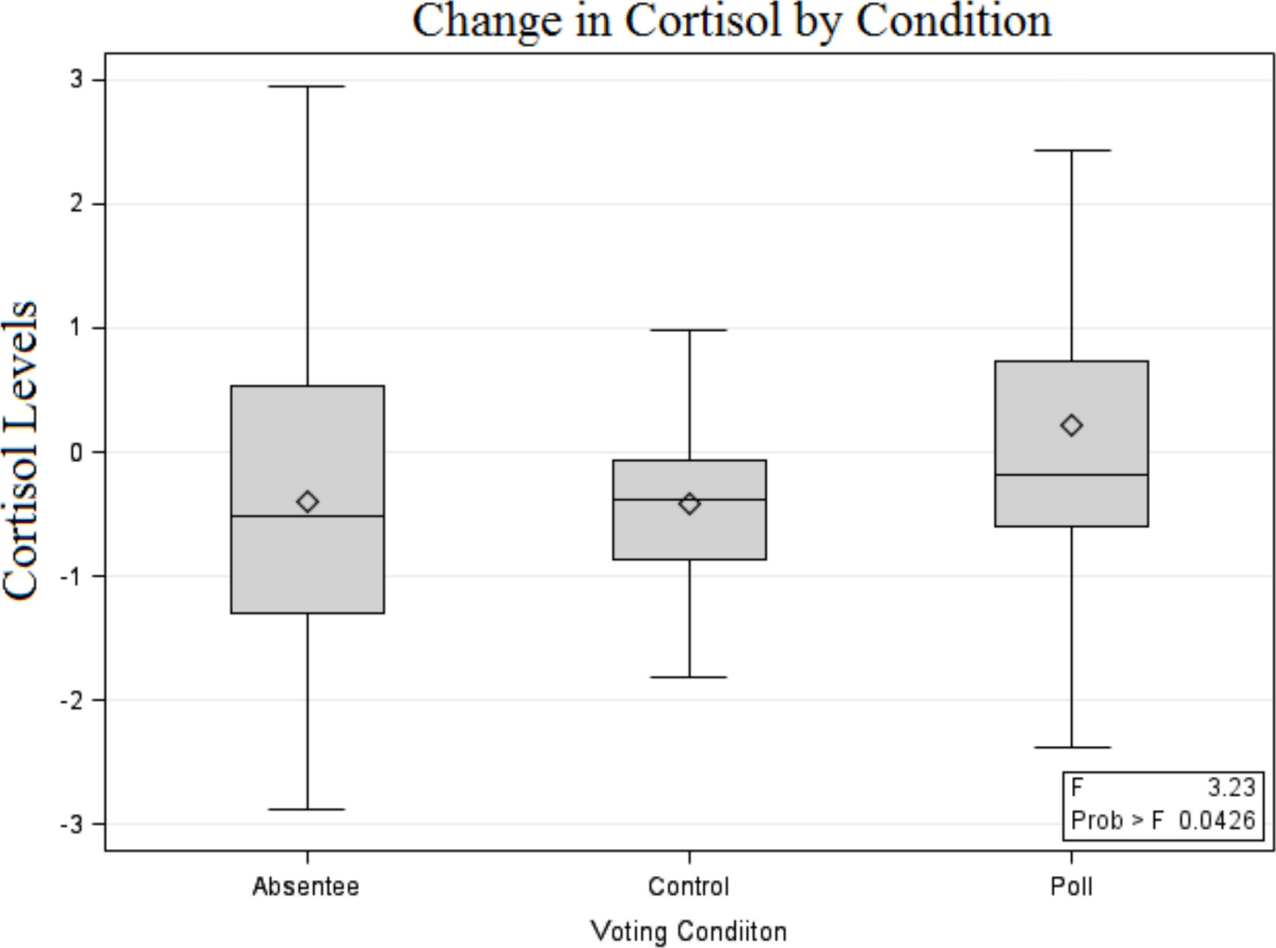
Cortisol Change by Voting Modality.

The first direct test of our hypothesis is a simple one-way ANOVA to examine mean differences in cortisol change across group. Mean cortisol changes in both the control condition (mean = -.42 ng/ml, SD = 1.18) and the absentee condition (mean = -.40 ng/ml, SD = .22) were negative. This is the pattern expected if the target behaviors were having no impact on diurnal cortisol rhythms. In the poll condition, however, cortisol change was positive, with a mean increase of. 21 ng/ml increase (SD = 1.39). Differences in these means were significant, F(2,130) = 3.2, p <.05. Post hoc tests suggested the poll condition is different from the control condition (Fisher LSD p = .03, Tukey HSD p = .07, Dunnetts t with control as reference category p = .05), but that the absentee condition was not (Fisher LSD p = .94, Tukey HSD p = .99, Dunnetts t = .99). There is also some suggestion that the poll condition is different from the absentee condition (Fisher LSD = .03, Tukey HSD p = .08). The basic pattern of these results is reported in Fig 1.

In addition to the ANOVA we also ran several regression models to get point estimates of the impact of the poll condition on cortisol under a variety of control conditions. These analyses are reported in Table 3. The first model is essentially the ANOVA just described reported in a regression format. Here the dependent variable is cortisol change and the independent variables are dummies for the poll and absentee condition. So in this model, the intercept is the estimate for the control condition as the excluded reference category. The coefficient for the absentee condition is zero and statistically insignificant, indicating cortisol change is this condition is indistinguishable from cortisol change in the control condition. The coefficient for the poll condition is. 62 and significant, indicating that mean cortisol change in the poll condition is. 62 ng/ml higher compared to the control condition, i.e. basically the same absolute difference reported between the mean cortisol change in the control (-.41 ng/ml) and poll (.21 ng/ml) conditions reported above.

**Table 3:** Estimates of Cortisol Level Change by Experimental Condition.

Variable	Model 1 (DV = Cortisol Change)	Model 2 (DV = Cortisol Change)	Model 3 (DV = Cortisol Level)
**Intercept**	-.40* (.20)	-.04 (.97)	-1.21 (.18)
**Absentee Condition**	.00 (.291)	.09 (.28)	.07 (.54)
**Poll Condition**	.62* (.28)	.76* (.27)	1.25* (5.2)
**PANAS**		-.01 (.07)	.12 (.14)
**Gender**		.08 (.23)	-.19 (.44)
**Age**		.00 (.00)	.01 (.01)
**Education**		.13 (.07)	.17 (.14)
**Income**		-.23* (.07)	-.43* (.14)
**Pre-Treatment Cortisol Level**		-.15* (.05)	1.10* (.10)
**N**	133	123	123
**F**	3.16*	3.52*	16.87
**Adj. R-2**	.03	.14	.51

*p <. 05, unstandardized coefficients (standard errors) reported

The second model has the same dependent variable and dummies for absentee and poll conditions, but also includes all of the equivalency control variables listed in Table 2. Here the intercept is not the estimate for the control condition (it is the estimated mean change in cortisol when all variables in the model are zero), but the other two condition dummies still represent point estimates of differences from the control condition. As can be seen, the estimate for absentee condition remains substantively and significantly zero. The coefficient for the poll condition, however, actually increases in the presence of controls, suggesting the mean cortisol change in the poll condition is. 72 ng/ml higher when accounting for the controls. Of those controls, only two are significant, income and pre-treatment cortisol levels. The coefficient for pre-cortisol levels is-.15, which suggests that for every 1 ng/ml of cortisol recorded before treatment, average cortisol change after treatment is-.15 ng/ml, controlling for all other sources of variance in the model.

The final model includes the same independent variables as the second model, but here the dependent variable is post-treatment cortisol levels rather than cortisol change. In short, this model represents the results of a pre/post experimental design that also controls for a variety of potential confounds. Again, we see a significant impact of the poll condition. The model estimates that in the poll condition, on average post-treatment cortisol levels (as opposed to change in cortisol levels) were 1.25 ng/ml higher than in the control condition. As expected, pre-treatment cortisol levels are a strong predictor of post-treatment cortisol levels (the measures were, after all, taken only an hour apart) and income is again a significant contributor to the model. Model 3. Though our primary interest is on changes in cortisol rather than cortisol levels, Model 3 is clearly suggests that participants in the poll condition end up with significantly higher cortisol levels than in the other groups after treatment, even though there were no such differences in pre-treatment cortisol levels (see Table 2).

The analyses presented in Table 3 essentially confirm the inference suggested by Fig 1. Even in the presence of a variety of controls, the poll condition is consistently associated with higher cortisol. Essentially, our analyses find the poll condition is significantly different from the absentee and control conditions, but that the absentee and control conditions are not significantly different from each other.

## Conclusion

Previous research on neuroendocrine function and politics has focused primarily on the tendency of pleasing or displeasing election outcomes (or anticipated outcomes) to affect cortisol and testosterone levels or on the correlation of endocrine levels and particular types of decisions, such as those that are aggressive [2,3,16–18]. Our concern here is different. We wanted to determine whether voting at the polls is measurably more stressful than voting at home.

Our data certainly seem to suggest that this is indeed the case. There are some caveats to our study. Collecting and processing the saliva samples necessary to obtain cortisol readings is more involved than conducting surveys and as a result sample sizes in work such as ours tend to be relatively small. In addition, going at the polls may trigger more direct thoughts about the election or other explicitly political experiences (e.g. pollsters or others asking about voting choices) that could potentially elevate cortisol levels. Our findings, in other words, are suggestive rather than definitive. Nonetheless, the population of our cells (47, 44, and 42) is substantial for experimental work and the overall N of 133 is larger than many physiological studies. As is always the case, more work is needed to replicate and extend these results, but in this particular sample the findings hold up under significant scrutiny and a battery of statistical tests. With random assignment and experimental controls, and even with additional statistical controls included on top of that, stress—at least as measured by cortisol changes—is consistently greater when people travel to the polls than when they vote at home.

Moreover, the stress accompanying voting at the polls is not merely the result of people needing to get out of their easy chairs in order to drive somewhere, stand in line, mingle with others, and make a choice. If this hypothesis was correct, traveling to a convenience store to select a magazine (the control group) should equally elevate cortisol levels as much as traveling to the polls to select governmental officeholders. Instead, voting at the polls is significantly more likely to elevate cortisol levels than either going to a convenience store or voting at home. Apparently the stressful element of voting at the polls is not just going out in public and not just making an important political choice. It is the combination of going out in public *and* making an important political choice that makes political engagement stressful. Voting at the polls elevates cortisol in a way that voting at home does not.

The role of stress in voting has been understudied. The research presented here suggests voting modality co-varies with stress response. In connection with the previous work on cortisol and voting, this research has potential implications for increasing political participation among highly stressed individuals who avoid the inevitable social conflict of political engagement. Following up on these implications, however, is but one promising avenue for future research. Among other aspects of political engagement and voting stress responses of the neuroendocrine system may also influence voting error rates, the incidence of spoiled ballots, and the overall quality of voting decisions. We have only begun to scratch the surface of the physiology of voting decisions.

## Supporting Information

S1 File Survey Items. (ZIP)

S2 File Methods. (ZIP)
